# The Bitter Cry of the General Practitioner

**Published:** 1888-07-28

**Authors:** 


### The Hospital

A Weekly Institutional Journal of

Science, Medicine, Nursing, and Philanthropy.

*For Hospitals, Asylums, Medical Practitioners, Students, Nurses, Families, Parishes, Congregations, and the Charitable Public.*

Vol. IV. No. 96.] [July 28, 1888.

### The Bitter Cry of the General Practitioner.

The average Englishman is a stolid kind of animal. He does not generally cry out before he is hurt, or for a small matter. When he does cry he takes care to make himself heard, and usually manages to make himself felt as well. The Englishman, in fact, is the kind of man who possesses a full share of the capacity of taking care of himself. It is a good wearing faculty that of self-reliance. Too much submissiveness is not very courageous nor very manly. It may be a good thing when stern necessity is at the helm. But absolute necessity is not always, or even very often, at the helm. There is generally some way out of a difficulty if a man have but the courage to find it.

The general practitioners of medicine, who practically constitute the bulk ot that humane profession, have long been hard driven. Hard driven by mean and poorhearted creatures within their own ranks, who, rather than act as qualified assistants to other and abler men, cut down their fees and their charges for medicine to the lowest possible level compatible with the avoidance of starvation; hard driven also, and, if possible, even more, by the hospitals which spring up on all hands like flies in a hot summer, and which, being helped by public charity, compete with the general practitioner on such advantageous terms that he has no possible chance of keeping up with them. The result is a hand-to-hand struggle with the difficulties of life which makes many a doctor's existence a grim tragedy from first to last.

Now our sympathies are entirely with the family doctor. He is, as a rule, a straightforward man—a man of education; he has been accustomed to the decencies and refinements of life from his birth; he does more personal and anxious work for less pay than perhaps any other man, and, in addition, he does, as a rule, as much work as would amount to a sixth of his income annually for nothing. Besides all this, he has to stick to his post day and night as no other man of any kind has to do. He is not merely a worker but a watcher, not merely a soldier by day but a sentinel by night. But yesterday the writer heard of a general practitioner, a noble man, who because he has to support the children of his dead brother has never married himself. He is now a white-haired man of only middle age; a man broken down by work and not by vices. Periodically he has to be long weeks absent from his practice at great expense and professional loss because the strain is too much for him. Speaking to a professional brother's wife he said, "A footfall on the pavement after I am in bed fills me with terror." What can compensate a man for a life thus spent? What is the material reward which the world offers to its patient and enduring medical heroes? A pittance, and often a grudging pittance, such as a foreman in a workshop would think an inadequate return for his services. But when even this pittance is snatched at by greedy competitors within his own ranks, and half of it stolen by hospitals bodily, what wonder is it that the doctor's soul should be filled with bitterness and his tongue with railing?

Dr. Page Hentsch and others are taking up cudgels resolutely on behalf of general practitioners. They are endeavouring to rally their illused brethren to a common standard. They think they have now discovered and localised a concrete foe in The Hospitals Association. Let us say authoritatively that they are mistaken. The Hospitals Association has not committed itself to the Pay System at hospitals in any form whatever, nor is it the least likely to do so. The Association is a deliberative body with the broadest possible sympathies. It offers a platform to all the friends of hospitals, and even to their opponents. Its one object is to make the hospitals effect the utmost possible good at the least possible cost, and with the smallest amount of injury to other people. So far from The Hospitals Association, or this journal, being an enemy to the general practitioner, each of them is one of his staunchest friends. If general practitioners as a body would make a point of becoming members of The Hospitals Association, they might by its powerful instrumentality greatly diminish, and perhaps entirely remove, every ground of reasonable complaint they have against the hospital system.

What may properly be affirmed against general practitioners in this matter is that they do not seem to understand the question at issue. They refuse to look at the facts. They will not face the difficulties. They feel hurt, and they cry out; but they do not cry intelligently. Like the Scotchman who used profane language, they "swear at large." But no good will ever come of such beating the air as this. If the hospitals are to be checked in the competition which is so ruinous to medical men, it will not be by vague charges and bitter cries, but by intelligent, combined, and resolute action.

What are the broad facts simply stated? These. There are large numbers of poor people in town and country who need medical assistance but cannot pay for it. There is a public conscience which insists that those poor people shall have medical assistance in spite of their inability to pay for it. The problem is to limit the gratuitous assistance to the really necessitous class. The giving of charitable assistance by hospitals to this class, and to it exclusively, is all clear gain to the doctor. For if the members of the class can pay nothing at all, any service a medical man may render in work or medicine is so much entirely given away, so much dead loss. There is then and can be no quarrel between the doctor and the hospital with regard to the really needy. The true bone of contention is the class immediately above the needy.

Now, strange to say, this bone is not really wanted by the hospitals, but is wanted by the general practitioners; and yet it is often found at the hospitals. How is this? There must be a reason for it. What is the reason? "Because the hospitals treat for nothing," say the practitioners, "whilst we make a charge." Precisely. Then what is the remedy that suggests itself, at any rate to common sense? Is it not this: that the hospitals should charge as much as the general practitioners, and so equalise the conditions of the competition? "No," say the general practitioners, "that would only make matters worse." Why, then, would it make matters worse? Is the hospital waiting-room more comfortable than the private practitioner's? Is the attention given to patients at the hospital more prompt and polite than at the private practitioner's? Is the skill of the average out-patient physician superior to that of the careful and honest private practitioner? Every one of these questions we answer in the negative. We firmly believe that if the scale of fees charged at the out-patient departments of hospitals to the non-necessitous were brought up to the level of that of the respectable general practitioner the result would be the practical emptying of those departments of all but the really necessitous poor.

But though that would be an effective remedy for the ills of which family practitioners complain, it is not the only remedy, and it may not even be the best. There is one other. The hospitals can refuse altogether to treat any who are above the necessitous class on any terms whatever. That, it seems to us, would lead to the same result as a just and thoroughly-applied Pay System; that is, it would drive non-necessitous patients to the consulting-room of the private practitioner. But though the latter course would have the same effect as the former, and for the purposes of a merely academic discussion offers a plausible alternative, the really important question is which of the two would be found most effectual in practice? This is the crux of the whole difficulty. It seems to us, after a careful consideration of the probabilities, that the Pay System would be entirely effectual in practice, whilst what may be called the "exclusion" system would as completely fail. It is impossible at the end of an article to state at length the reasons for this conclusion. The main line of reasoning would be that by the "exclusion" system the hospitals have nothing to gain and everything to lose. They are asked by it to undertake critical, laborious, and unpleasant investigations, not in their own interests, but in the interests of the general practitioners. Knowing human nature as we do, and especially official human nature, we feel certain that the exclusion system will never be applied at hospitals by hospitals themselves. It must either be done by an independent organisation or some other alternative must be adopted. What other alternative has ever been suggested except the Pay System?

